# Studying the Effect of Amino Acid Substitutions in the M2 Ion Channel of the Influenza Virus on the Antiviral Activity of the Aminoadamantane Derivative *In Vitro* and *In Silico*

**DOI:** 10.34172/apb.2021.079

**Published:** 2020-07-15

**Authors:** Timur Mansurovich Garaev, Artyom Irorevich Odnovorov, Alexander Aleksandrovich Lashkov, Tatiana Vladimirovna Grebennikova, Marina Pavlovna Finogenova, Galina Kadymovna Sadykova, Alexei Gennadievich Prilipov, Tatiana Anatol'evna Timofeeva, Sergey Vadimovich Rubinsky, Svetlana Nikolaevna Norkina, Marina Mikhailovna Zhuravleva

**Affiliations:** ^1^Federal State Budgetary Institution «National Research Centre for Epidemiology and Microbiology named after the honorary academician N.F.Gamaleya» of the Ministry of Health of the Russian Federation (N.F.Gamaleya NRCEM), 123098, Moscow, Russian Federation.; ^2^Peoples Friendship University of Russia (RUDN University), Ministry of Education of the Russian Federation, 117198, Moscow, Russian Federation.; ^3^FSRC «Crystallography and Photonics» RAS, Leninskiy Prospekt 59, 119333, Moscow, Russia.

**Keywords:** Antiviral, Drug resistance, Influenza virus, M2 proton channel, Molecular docking, Reverse genetics

## Abstract

***Purpose:*** The aminoadamantane derivative of L-histidyl-1-adamantayl ethylamine hydrochloride (HCl*H-His-Rim) has showed a high inhibition level against influenza A virus strains *in vitro*. The aim of this work is to search and establish evidence of the direct effect of the drug on influenza A virus proton channel M2.

***Methods:*** The compound HCl*H-His-Rim was obtained by classical peptide synthesis methods. Influenza A virus mutants of A/PuertoRico/8/34(H1N1) strain were obtained by reverse genetics methods. The mutant samples of the virus were cultured on chicken embryos with a virus titer in the hemagglutination test. ELISA was carried out on Madin-Darby canine kidney (MDCK) monolayer cells when multiplying the virus 10^-4^-10^-6^. The binding stability of HCl*H-His-Rim was compared to those of M2 (S31N) and M2 (S31N_A30T) channels by molecular dynamic (MD) modeling. The calculation was performed taking into account the interaction with the model lipid bilayer (1-palmitoyl-2-oleoyl-sn-glycero-3-phosphocholine) in the presence of water molecules in accordance with the three-center model.

***Results:*** It was found that HCl*H-His-Rim is a direct action drug against influenza A. The most likely conformation of drug binding to target protein has been shown. It has been found that the A30T mutation reduces the binding energy of the drug, and the results obtained *in vitro* have confirmed the data calculated *in silico*.

***Conclusion:*** The mechanism of action of HCl*H-His-Rim is directly related to the suppression of the function of the proton channel M2 of influenza A virus.

## Introduction

The M2 proteins of Influenza A virus are transmembrane proteins that form proton-selective channels in the lipid membrane. M2-channel is a homotetramer structure assembled from four subunits of the M2 protein. Polypeptide chains with a length of 97 amino acid residues are partially helical in the region of the transmembrane domain.^1^ Proton conductivity of M2 channels is necessary for virus replication. Proton-selective M2 channels regulate pH inside the viral particle during virion penetration into the endosome of the cell by acidification the inner space of the viral particle, which results in dissociation of the matrix protein M1 complex with ribonucleotide and leads to the release of genetic material into the cytoplasm of the host cell.^2,3^ Another important role of M2 protein appears at a later stage during viral assembly, when newly synthesized viral proteins are transported to the cell membrane surface. At low pH values, the virus proteins, in particular, hemagglutinin (HA), can undergo premature and undesirable transformation; in this case the M2 proton channels pump hydrogen ions from the Golgi complex to maintain a sufficiently high pH value and preserve the transport form of the newly formed HA.^4,5^

The M2 viral protein channel is a target for antiviral therapeutic drugs, namely amantadine and rimantadine, which belong to the class of adamantane compounds. Aminoadamantanes have been used for the treatment and prevention of influenza A since the 1970s. Their economic and synthetic availability made them ideal for treating seasonal influenza epidemics around the world. However, because of the widespread use of these drugs, the influenza A virus, which has a high mutation rate, underwent genetic modification making it resistant to the action of adamantane-type drugs. In laboratory strains, scientists observed various mutations in the transmembrane domain of the M2 channel; however, the wild type is characterized by three main mutations, the amino acid substitutions of the serine residue with asparagine in 31 positions of the M2 protein being considered critical for the ability of aminoadamantanes to block the functions of the channel.

**Figure 1:**
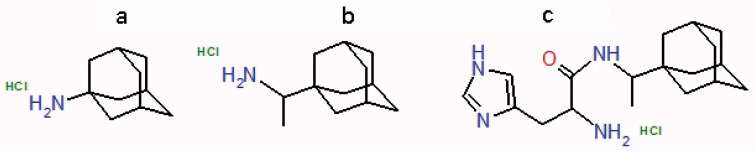
Structure of aminoadamantans - amantadine (a), rimantadine(b) and amino acid derivative of rimantadine L-histidil-1-adamantayl ethylamine (HCl*H-His-Rim) (c).

The Centers for Disease Control and Prevention (CDC), in association with WHO, recommend no use of rimantadine and amantadine (Figure 1) for the treatment of influenza because their resistance has exceeded 90%.

The aim of this work is to study the mechanism of antiviral effect of HCl*H-His-Rim compound (Figure 1) on the proton-conducting channel M2 of influenza A virus through the identification of amino acid substitutions in the pore of M2 channel, affecting the antiviral activity of the compound and analyze the most significant mutations by molecular docking methods.

## Materials and Methods

### Compound. HCl*H-His-Rim

The formation of a peptide bond between the carbocycle of rimantadine and di-*tert*-butyloxycarbonyl-L-histidine was carried out in a single stage under the conditions of mixed anhydride method in the equimolar ratio according to the procedure described.^6-8^ The removal of protective *tert*-butyloxycarbonyl groups was carried out in a solution of 4 N HCl in ethyl acetate followed by vacuum drying to a dry foam. Yield: 92%. M.p. 210°С (decomposition); [α]^20^_D_ = +6° (c = 1; C_2_H_5_OH), IR: υ(NH) 3248 cm^-1^; υ(NH^im^) 3139 cm^-1^; υ(C=O) 1672 cm^-1^. *m/z* found [M + H]^+^: 317,746; [M + Na]^+^: 339,761; calculated M (C_18_H_28_N_4_O) 316,441 (Figure S1). ^1^H NMR (D_2_O, ppm): 8.81 (s, 1H, NH-CH=NH), 7.52 (s, 1H, NH-CH=C), 4.79 (H_2_O (HDO) + NH-atoms), 4.31 (t, 1H, CH2-CH-NH_2_, 8 Hz), 3.49 (q, 1H, NH-CH-Ad, 7Hz), 3.40 (d, 2H, His-CH2-CH, 8 Hz), 1.90 (s, 3H, Adamantyl β-H), 1.60 (dd, 6H, Adamantyl γ-H, 56 Hz, 11 Hz), 1.22 (dd, 6H, Adamantyl α-H, 41 Hz, 11 Hz), 1.04 (d, 3H, CH_3_-CH-Ad, 7 Hz);^13^C NMR (D_2_O, ppm): 166.9 (C=O), 134.1 (NH-CH=NH), 126.4 (NH-C=CH), 118.3 (NH-C=CH), 54.7 (CH3-C-Ad), 52.5 (CH_2_-CH-NH_2_), 37.7 (Adamantyl α-C), 36.3 (Adamantyl γ-C), 35.1 (CH_2_-CH-NH_2_), 27.8 (Adamantyl β-C), 26.1 (Adamantyl C-CH-NH_2_), 13.1 (CH_3_-C-Ad) (Figure S2).

### Molecular docking, dynamics and free energy calculations

The quantum-mechanical model of the ligand was generated in HyperChem 8.0.8 software product by Hypercube (https://hyper.com/).

The structure of the transmembrane domain M2 S31N from RCSB Protein Data Bank (structure code 2KIH) was taken as a target for docking to be performed. The docking of the ligand model into the protein channel was carried out using an ant colony optimization algorithm in PLANTS program.^9^ For the ligand, flexible docking with the possibility of *cis*-to-*trans* transition via an amide bond was used. For the initial assessment and ranking of docking results, the ChemPLP scoring function was used.^10^ To study the binding stability of HCl*H-His-Rim in conformations selected from molecular docking solutions with M2 S31N and M2 S31N_A30T, molecular dynamic (MD) modeling of this complex was performed in GROMACS software package (version 2018.8).^11^ Ligand models with arrangement of partial electric charges of atoms, parameters of Van der Waals interactions, stiffness constants for valence bonds, valence and dihedral corners were processed using the CGenFF WEB service.^12^ The layout and parameterization of MD-systems of protein-ligand complexes in lipid bilayer was carried out in the CHARMM-GUI: Membrane Builder WEB service^13^ using a set of CHARMM^14^ full atomic force fields of version C36m.^15^ The model lipid bilayer consisted of 1-palmitoyl-2-oleoyl-sn-glycero-3-phosphocholine molecules. The model was built in a hexagonal box with dimensions *X* = *Y* = 100 Å. The parameter *Z* was selected automatically based on the condition that the distance from lipid atoms to the upper and lower box should be at least 22.5 Å. These volumes were filled with water molecules in accordance with the transferable intermolecular potential with 3 point (TIP3P) model and with the addition of neutralizing Na^+^ and Cl- ions of 0.1M concentration. The same concentration of counter ions was introduced into the ligand-solvent system.

The simulation was carried out by the molecular dynamics method. To integrate Newton’s equations of motion with a step of 2 fs, a leap-frog algorithm was used. The path length was 20 ns. The long-range electrostatic interactions were calculated by the particle-mesh-Ewald method (PME) using the cubic interpolation function. To describe the van der Waals interactions, we used the force-switch smoothing function in the range from 10 to 12 Å. The pressure in the system was controlled by the Parrinello-Rahman pressure coupling^16^ at the level of 1 bar. The temperature of the MD-system was kept constant by the velocity rescale temperature coupling^17^ at 310 K. Molecular dynamics procedure was preceded optimizing atomic parameters by the method of steepest descent and simulation of the system with a position restraints of heavy protein and ligand atoms in canonical (NVT) ensemble and isothermal–isobaric (NPT) ensemble (the Berendsen’s pressure coupling^18^ was used) according to Table S1.

The density map of the radial distribution of water averaged over the path of the MD simulation was constructed using the g_ri3Dc program.^19^

The relative affinity of ligand binding was determined by calculating the linear interaction energy^20,21^ (LIE) using the gmx lie program of GROMACS software package. To make this, the average energies of van der Waals and electrostatic ligand-water interactions in the ligand-water system were previously calculated during MD simulation of 20 ns. Protein-ligand MD-simulation trajectories were re-processed using shift potential at 12 Å cutoff to calculate the potential energy of Coulomb interaction between the ligand and the environment. The empirical constants α and β, which determine the contribution of van der Waals and electrostatic interactions to the estimation of the binding energy, were selected in accordance with Hansson et al^21^ and were equal for HCl*H-His-Rim: α = 0.18 and β = 0.5. The shift coefficient γ was taken equal to zero due to the impossibility to select it at this stage of the study.

The ligand-protein free energy of binding was calculated usingthe thermodynamic cycle described Aldeghi et al.^22^ The first stage consisted of collecting the derivative values of the system Hamiltonian by the Kirkwood coupling parameter^23^ (λ-parameter) (for Thermodynamic Integration (TI)) and the difference in the potential energy of the system between the current and other states (for Free Energy Perturbation (FEP)-based methods). For protein-ligand and protein-water systems, MD simulations were performed in the NPT ensemble for 2.4 ns using a leap-frog integration algorithm of stochastic dynamics equations for each set of λ-parameters. In order to retain the ligand in the binding region during its decoupling, additional intermolecular restrains were introduced as described Aldeghi et al.^22^ For the protein-ligand system, λ changed according to the sequence of λrestr (0.0, 0.01, 0.025, 0.05, 0.075, 0.1, 0.15, 0.2, 0.3, 0.4, 0.6, 0.8, 1.0) and λ_coul_ (0, 0.2, 0.4, 0.6, 0.8; 1); λ_VdW_ (0, 0.05, 0.1, 0.2, 0.3, 0.4, 0.5, 0.6, 0.65, 0.7, 0.75, 0.8, 0.85, 0.9, 0.95, 1.0). In the case of the ligand-water system, coupling parameters changed only for the Coulomb and van der Waals ligand-water interactions: λ_coul_ (0, 0.25, 0.5, 0.75, 1); λ_VdW_ (0, 0.05, 0.1, 0.2, 0.3, 0.4, 0.5, 0.6, 0.65, 0.7, 0.75, 0.8, 0.85, 0.9, 0.95, 1.0). The correction for the energy of the restrains introduced for the ligand-water (ΔG_solv_rest_) was calculated analytically using the formula reported Boresh et al.^24^ Prior to the main stage of MD simulations, in addition to the general equilibration of the system described above, for each value of the coupling parameter λ, energy minimization and equilibration were carried out with restrictions imposed on the positions of heavy protein and ligand atoms in the NVT and NPT ensemble for 100 ps. Van der Waals interactions were calculated using the Lennard-Jones (LJ) “soft core” potential. The PME method was used to calculation both long-range Coulomb and van der Waals interactions.

Data of FEP MD simulations were processed in a program written in Jupyter Notebook using alchemlib and pymbar libraries. The first step was the parsing of xvg files with the extraction of reduced potentials using alchemlyb.^25^ The second step was to select uncorrelated values of reduced potentials (U_ij_) with cutting off the non-equilibrated region of trajectories using the detect equilibration function of the pymbar library.^26^ For each window, the difference of reduced potentials between current and the neighboring window was used to calculate statistical inefficiency. In the third step, the statistically independent values of U_ij_ were processed using the Multistate Bennett Acceptance Ratio (MBAR) method.^27^ The correctness of the calculations was controlled by the overlap matrix between the λ-states, as well as by comparing the obtained ΔG values with the values obtained by other methods: Exponential averaging,^28^ Bennett Acceptance Ratio,^29^ TI.^30^

Since the total charge of ligand atoms in the MD simulation was +1 e, it was necessary to calculate the corrections for Ewald summation and periodic boundary conditions. They were calculated using analytical scheme as described Rocklin et al.^31^ The protein charge was completely compensated by counterions and effective Q_P_ = 0. The box was converted to orthorhombic to simplify these calculations. The average linear box parameters *t* were 9.682 × 8.384 × 10.395 nm for the protein-ligand system, and 3.433 × 3.237 × 2.804 nm for the ligand-solvent system. The difference in linear parameters between the different complexes was less than 5%. Roughly the same fluctuations in the parameters were observed as a result of the pressure coupling algorithm work in the NPT simulation. 1/(4πε_o_) was assumed to be 138.93545585 kJ nm e^-2^ mol^-1^, ε_s_ for TIP3P water was 82, ρ_310K_ = 993 kg/m^3^, and γ_S_ = 0.0764 e nm^2^. Residual integrated potential (RIP) correction was calculated using APBS-mem^32^ and APBS.^33^ In calculating RIP and discrete solvent effects (DSF) corrections, the following parameters of the model lipid bilayer were used: D_M_ = 4.51 nm, D_HEAD_ = 0.9 nm.^34^ The relative permittivity of protein and solvent were ε_s_ = 2, ε_H_ = 80.

### Plasmid transfection

The source virus and mutants based on it were obtained by reverse genetics. A system of eight plasmids containing DNA copies of genome segments of the influenza virus strain A/PuertoRico/8/34 (H1N1) was used in this work.^35^ Nucleotide substitutions for mutant production were introduced using the commercial QuikChange XL Site-Directed Mutagenesis Kit (“Stratagene”, USA). Plasmid transfection was performed in mixed culture HEK-293T-MDCK.^36^

Viruses obtained by plasmid transfection were passivated to the allantois cavity in 10-day-old chicken embryos. Infected chicken embryos were incubated for 48 hours at 37°C and then cooled overnight at 4°C. Virus-containing allantois fluid was collected under sterile conditions and the virus content was evaluated as a titer obtained in the hemagglutination reaction^37^ (HGA) expressed in terms of the number of hemagglutinating units (GAUs). The preparations were stored at -80°C.

### Polymerase chain reaction and sequencing

Viral RNA was isolated from the virus-containing allantois liquid using the RNeasy Mini Kit (Qiagen). Reverse transcription and PCR were performed with primers to the influenza A virus *M* gene. Amplification products were purified using the QIAquick PCR Purification Kit (Qiagen). Sequencing was performed using the DNA ABI Prism 3130 automatic DNA sequencer (Applied Biosystems) and the BigDye Terminator v3.1 Cycle Sequencing Kit (Applied Biosystems). Nucleotide sequences were analyzed using the DNASTAR Sequence Analysis Software Package (DNASTAR Inc.).

### Determination of antiviral activity

The antiviral activity of the test compound (HCl*H-His-Rim) against influenza A/PuertoRico/8/34(H1N1) was determined using immunoassay (EIA) in MDCK cell culture, during the dilution of the virus 10^-4^-10^-6^ breeding. The compound was added in concentrations of 10.0 µg/mL (31.5 μM) at the same time as the cell monolayer was infected. The test compound and dilution of the virus were prepared in a medium supplemented with TPCK trypsin (2.5 µg/mL) in tests using influenza virus A/PuertoRico/8/34(H1N1) strains and mutants of this virus. The plates were incubated for 24 h at 37°C, after which the reaction was stopped by treating the cells with 80% acetone in a phosphate buffer. Cellular EIA was performed according to the previously described method.^38,39^ The percentage of viral activity inhibition of the compound was determined as the ratio of [experimental optical density of OD_450_ – OD_450_ cell control / OD_450_ virus control – OD_450_ cell control] × 100%.

## Results and Discussion

The development of new therapeutic agents is an urgent task due to the fact that classical direct-acting drugs such as rimantadine and amantadine are not effective because of drug resistance formed. The function of the ion channels can be blocked by small inhibitor molecules (in particular, adamantane-series preparations), which leads to a significant decline in the reproduction of viral particles. The action mechanism of the aminoadamantane derivative is the subject of this study.

The mechanism of inhibition of amantadine and rimantadine has been intensively studied during the last two decades. Before the appearance of high-resolution crystallographic structures, the drug binding site was predicted from the position of mutations that led to the emergence of resistant strains of influenza A virus. Mutations that are responsible for resistance to amantadine or rimantadine are found in the following protein M2 positions: 26, 27, 30, 31, 34 and 38.^40-42^ The authors^42^ generalized all hypotheses known to date regarding the binding sites of amantadine with the M2 protein. Most of these positions, including 27, 30, 31 and 34, are located on the inner surface of the M2 channel, which led to the hypothesis of drug penetration into the channel.

Studies^43^ showed that the formation of a classical hydrogen bond is not a prerequisite for the inhibitory effect on proton conductivity. There is an evidence of a mechanism in which the amantadine amino group does not interact with any of the M2 channel pore polar groups. For example, the S31A mutant was sensitive to rimantadine,^44^ which indicates that S31 does not play a decisive role in binding to the drug.

The inner surface of the M2 canal is composed mainly of non-polar amino acid residues, and the formation of reliable hydrophobic interaction with the ligand is sufficient for antiviral activity to occur. Duff et al^45^ showed that amantadine amino group can interact with the imidazole imidazole pairing of His37 residues; therefore the presence of an additional imidazole ring in the channel pore may lead to disruptions in the protonation process due to competitive reactions with 2HCl*H-His-Rim imidazole ring.

Thus, the main supposed mechanism of antiviral action of synthetic compound 2HCl*His-Rim is probably similar to that of amantadine on the M2 ion channel.^41^

Based on the predictions of primary molecular docking by reverse genetics methods,^46^ mutants of influenza virus A/PuertoRico/8/34(H1N1) containing amino acid substitutions in position V27, I28 and A30 were created.

The ability of the ligand to inhibit mutant virus replication in comparison with native A/PuertoRico/8/34(H1N1) influenza virus has been studied by computer simulation of ligand (2HCl*His-Rim) interaction with target protein (M2 channel) in the presence of amino acid substitutes V27A and A30T. Moreover, the mutation of A30T seemed most likely to achieve a decrease in the antiviral properties of the ligand compound due to the maximum difficulties in binding to the target protein. This is due to the large steric restrictions because of the increase in the volume of the side group. The V27A mutant binds optimally closer to the N-terminus of the channel, but in general the total binding energy was the same as that of the reference structure.

The situation with the double replacement of V27A + I28V was less favorable to achieve a result than A30T. Replacing the aliphatic group 2-butyl with the aliphatic group isopropyl could not significantly affect the stereometry inside the channel at position 28. The residue I28 was not turned into the channel, therefore it had little effect on the steric difficulties for the ligand, but could lead to a decrease in the strength of the hydrophobic interaction of the binding site with the adamantane core of the inhibitor molecule.

A reverse genetics system was used to prove the effects. This system is successfully used for scientific and applied research.^45^

The obtained mutants of influenza virus A/PuertoRico/8/34(H1N1) were tested *in vitro* for sensitivity to HCl*H-His-Rim. Influenza virus A/PuertoRico/8/34(H1N1) was sensitive to HCl*H-His-Rim; the compound successfully inhibited the replication of this strain at a concentration of 10.0 μg/mL (31.5 μM), the percentage of inhibition in the breeding of 10^-5^ virus was about 70% (Table 1). In turn, the three proposed mutants were less sensitive to the action of the compound than native A/PuertoRico/8/34(H1N1) influenza virus.

**Table 1:** Suppression of reproduction strains in comparison with the virus control in percent

**Virus**	**Virus reproduction suppression, %**		
**Virus dilution**	**10** ^-4^	**10** ^-5^	**10** ^-6^
A/PuertoRico/8/34(H1N1) (reference)	34.70 ±25.79	74.31 ±13.07	97.29 ±7.69
V27A	19.96 ±13.95	44.66 ±11.98	61.04 ±27.61
V27A + I28V	20.60 ±7.11	53.64 ±13.25	73.58 ±28.82
A30T	15.19 ±7.81	31.70 ±11.27	67.59 ±25.36

Table 1 shows the average of the twelve parallel experiments. The compound acquired the greatest loss of activity was mutant A30T. The mutants of influenza virus A/PuertoRico/8/34(H1N1) V27A and V27A + I28V were more sensitive to the compound, which showed comparable antiviral activity data.

For an in-depth study of the antiviral mechanism of the HCl*H-His-Rim compound, variants with native virus (reference) and mutant virus (A30T) were studied by molecular docking methods.

### Molecular docking, energy minimization and equilibration results

Based on the value of the ChemPLP scoring function, 10 docking solutions were selected for the reference structure and for the mutant channel structure A30T (Table 2). The solutions form distinct clusters according to the position of the ligand in the channel, taking into account the fact that the channel structure is symmetrical in the direction perpendicular to the bilayer plane (4th order axis). From each cluster of solutions, one solution was selected that was the most identical in position and conformation in the reference and A30T. Further the structures of the complexes were placed in bilayer, solvated and procedures of energy minimization and equilibrating were performed.

**Table 2:** Results of molecular docking

**Reference**	**A30T**		
**Rank**	**ChemPLP score**	**Rank**	**ChemPLP score**
1	-69.428	1	-73.548
2*(surface)	-68.463	2	-72.222
3	-67.901	3* (middle)	-70.381
4*(middle)	-66.756	4* (deep)	-69.802
5	-66.578	5	-68.693
6	-65.418	6*(surface)	-68.315
7	-65.232	7	-67.951
8	-64.954	8	-67.749
9* (deep)	-64.951	9	-67.289
10	-64.857	10	-66.783

(*-ligand conformations selected for later investigation).

**Figure 2:**
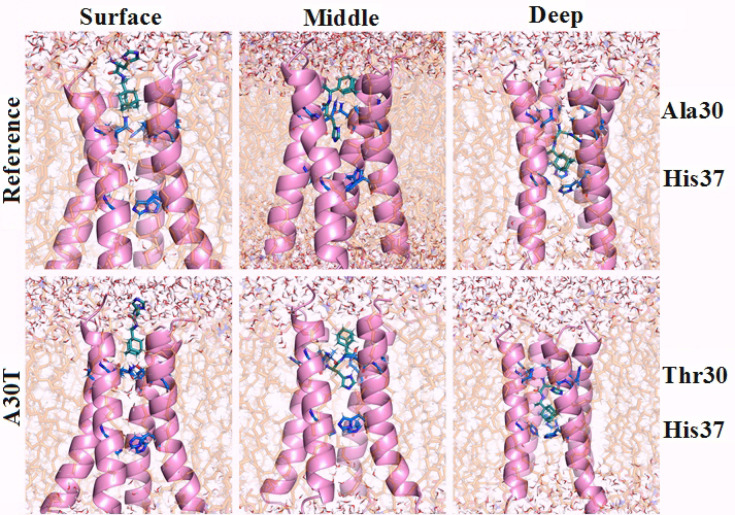
Structure of the outer (upper) part of the proton pump in combination with histidine rimantadine. Ligand conformations were detemined by molecular docking followed by the incorporation of complexes in the lipid bilayer, energy minimization and equilibration in NVT and NPT ensembles.

The first variant of possible ligand binding by a channel (surface) is characterized by the surface arrangement of the ligand (Figure 2). The adamantane skeleton is slightly submerged in the channel and is at V27 level. The histidine part of the ligand is adjacent to the side chain D24 and interacts with it through hydrogen bonds and electrostatic interactions. This part of the ligand also forms hydrogen bonds with water surrounding the entrance to the channel. It should be noted that after equilibration, the water molecules were found below the ligand H37.

The second variant (middle) is characterized by the adamantane core being between 27 and 30 amino acid residues, and the amino acid part interacts with the side group N31 and the main chain of 30 amino acid residues. At this stage of research, water molecules were not detected in the channel between V27 and H37.

The third option is the deep location of the ligand in the channel (deep). The imidazole ring of the histidine part of the ligand is located almost across the channel and is at the level of 30-31 aa. At the same time, the adamantane part of the ligand is located 0.35-0.4 nm higher than the plane of the H37 imidazole rings, which are essential for the functioning of the proton pump.^3^ Water molecules are located only above 30 amino acid residues.

### Free MD-simulation. The passage of water in the channel

**Figure 3:**
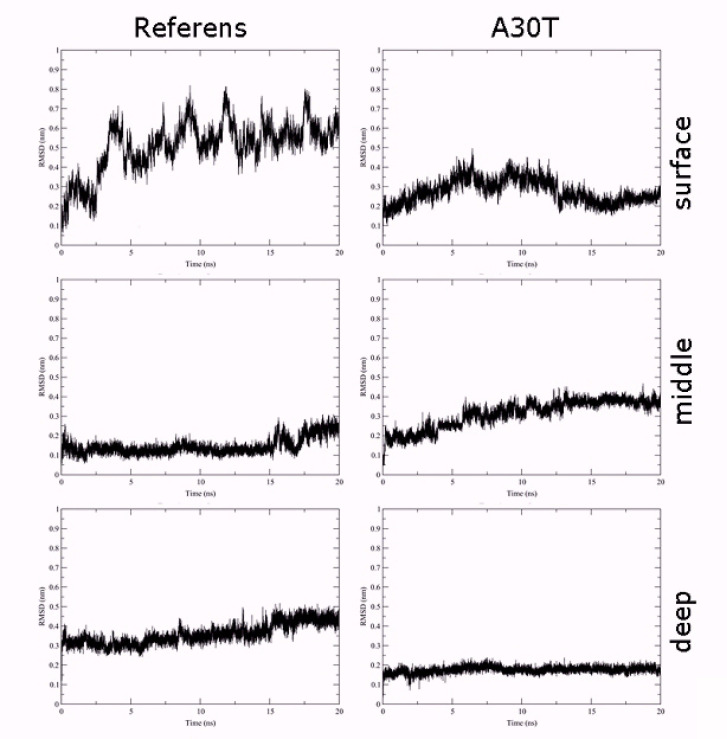
Standart deviation plots coordinates of the non-hydrogen ligand atoms with the respect to the initial position as a function of the MD simulation time simulation

Figure 3 shows the standard deviation plots of the non-hydrocarbon ligand atomic coordinates gen with the respect to the initial position as a function of the MD simulation time. As can be seen from the plot, the most stable conformation in both cases is deep. The ligand in the surface binding position is the most labile. In this conformation, there is a strong oscillation of the amino acid part of the ligand in the solvent, which surrounds the entrance to the channel, while the position of the adamantane component atoms exhibits less changes. In a medium binding situation, the position of the ligand atoms in the reference protein is more stable compared to the A30T mutant.

**Figure 4:**
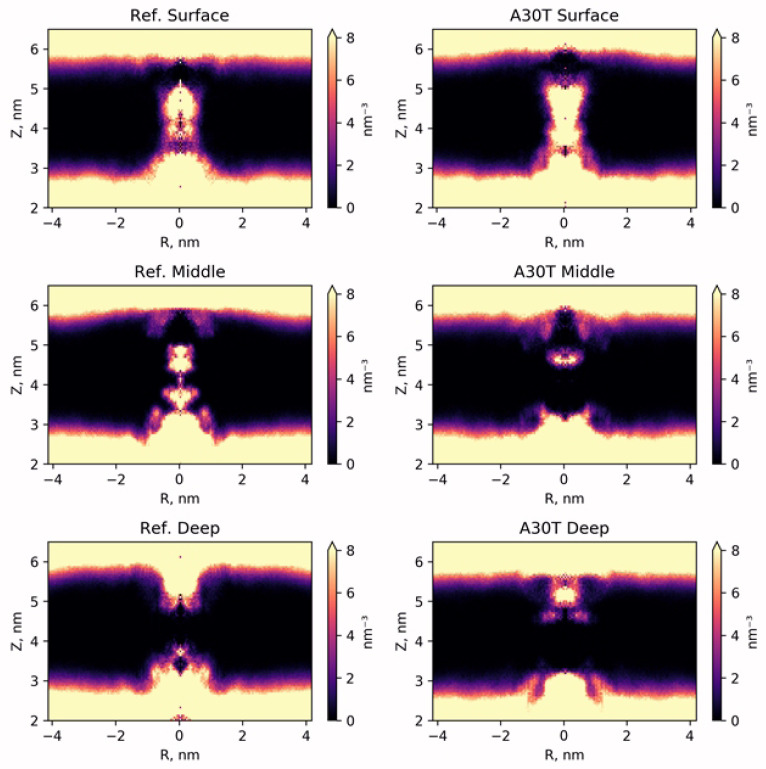
Map of the radial density distribution of water molecules averaged by DM-trajectory.

In order to study the distribution of water in the pore channel, a map of the dependence of water density on the distance to the center of the channel and the coordinates of the perpendicular plane of the membrane was created (Figure 4). In the case of surface fixation of HCl*H-His-Rim, both in the reference and in A30T, the density of water molecules in the channel is high both above and below H37. Apparently, the ligand in this position poorly blocks the water flow and, as a result, the activity of the proton pump. A significantly lower density of water molecules is observed when the ligand is in intermediate (mid) conformation. However, attention should be paid to the water-filled cavity between N31 and H37. At the previous stage of equilibration, no water molecules were observed in this volume. In case of long-time MD-simulation, water molecules penetrate from the inner side of the channel and pass through H37 and solvate the histidine part of the ligand, establishing a network of hydrogen bonds between the ligand and the channel wall. Significantly less diffusion of water into this region occurs from the upper part of the channel, since this diffusion is prevented by the adamantane hydrophobic fragment of the ligand. Thus, the water flow in the channel, in the case of the middle binding variant, is either insignificant or absent.

In the case of low ligand binding, water does not penetrate into the channel below 30 amino acid residue. From below, water flow is also not observed throughout the entire MD simulation trajectory. Thus, the ligand being in this conformation completely blocks the water current in the channel of both the reference structure and A30T mutant.

### Evaluation of the binding affinity of HCl*H-His-Rim conformations by the LIE Method. The role of water in binding the imidazole fragment of the ligand

**Figure 5:**
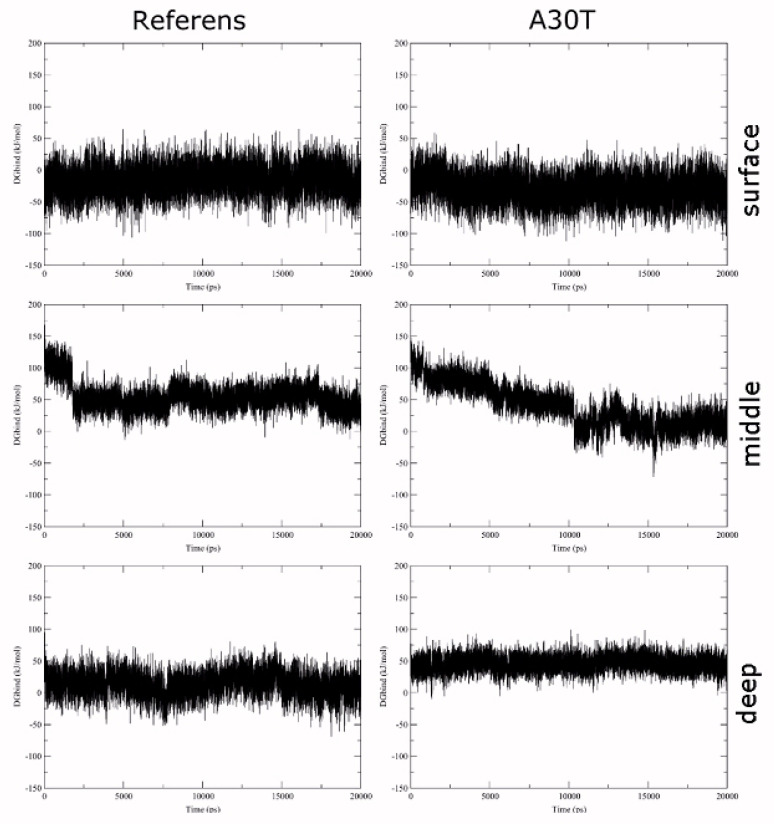
Dependence of the relative free binding energy of HCl*H-His-Rim on the time of MD-simulation

**Figure 6:**
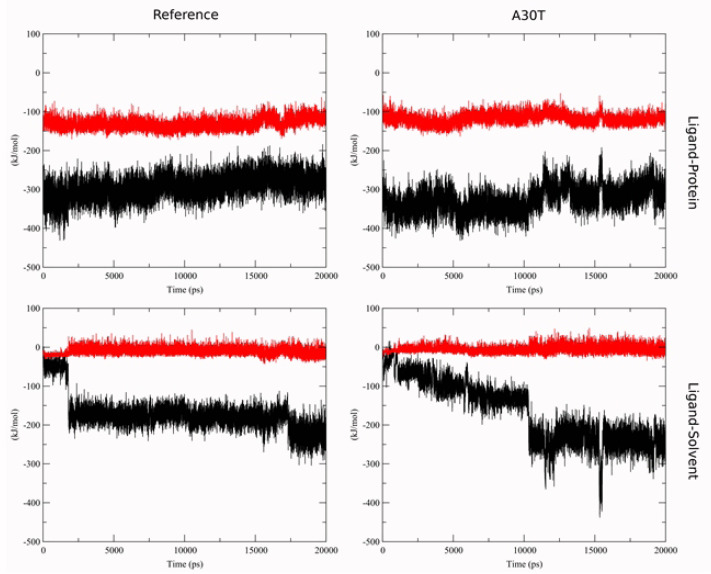
Short-range interaction energy for Coulumb (black) and Lennard-Jones term (red) for ligand-protein complex in middle conformation.

To estimate the affinity by the LIE method, the terms of the interaction energy of the ligand with the environment in the ligand-water system were first calculated: < U_Coul_> = -591.0 ± 3.0 kJ/mol, < U_lj_> = -77.8 ± 1.2 kJ/mol. These values were used to calculate the dependence of ΔG_LIE_ on the MD-stimulation time (Figure 5). The statistical parameters of the distribution of ΔG_LIE_ values are given in (Table S2). For surface binding, there is a larger scatter of ΔGLIE than for deep binding. It is explained by the higher ligand mobility in the surface conformation. In the middle conformation, a sharp change in the bind energy on the graph cannot be explained by the change of the ligand position in the binding site assumed. The short-range part of the Coulomb and Van der Waals energies of the interaction between ligand and protein and between ligand and solvent with counterions was analyzed with simultaneous viewing of the molecular plot. It was revealed (Figure 6) that in MD-simulation the energy of ligand-protein interaction undergoes no significant changes. At the same time, the interaction energy of the ligand with the solvent varies strongly and stepwise, both in the case of the average conformation of ligand-to-reference protein binding and with A30T. In the visual analysis of MD-simulation, it was found that these changes are associated with the penetration of water molecules into the cavity between N31 and H37. Water molecules pass mainly from the inner part of the membrane and then 3-4 water molecules constantly hydrate the histidine part of the ligand, establishing a network of hydrogen bonds between the ligand with water and amino acid residues of the protein. The structures of complexes with solvated ligand in the middle conformation were also chosen to calculate the free binding energy.

A sharp jump in binding energy is due to the transition of water molecules within the cut-off radius of the ligand atoms. The jump in the Coulomb term of the ligand-environment interaction energy in the A30T structure at 16 ns is due to a random character of approaching of the Cl- ion to ligand within the cut-off radius and is fast process.

The LIE data should be considered skeptically in terms of comparing possible ligand binding sites with each other, due to the significant difference in the hydrophobic properties of the ligand environment affecting the real value of the shift parameter γ.^47^ It should be noted that the Coulomb shift potential takes into account only interactions between particles at a distance not exceeding the cutoff radius. In addition, unlike the reaction field method, the dielectric permittivity value of the medium is not used here. The reaction field method is impractical to use, since the system is heterogeneous in terms of the dielectric permittivity value.

### Calculations of binding free energy

Table 3 shows the free energy of binding for ligand-solvent and ligand-receptor system obtained from MBAR method with electrostatics corrections and total ΔG_bind_. Total ΔG_bind_ was calculated by the formula: ΔG_bind_ = ΔG^prot^- ΔG^solv^, where

ΔG^x^ = ΔG^coul^ + ΔG^vdw^+ΔG^rest^+ΔΔG_DSF_ + (ΔΔG_NET_+ΔΔG_USV_) + ΔΔG_RIP_,

ΔG^coul^ is the free energy Coulomb interaction,

ΔG^vdw^ is the free energy of Van der Waals interaction, defined as Lenard-Jones potential,

ΔG^rest^ is the free energy coupling restraints (in the case of the ligand-solvent system, it is calculated analytically according to the formula described by Boresch S, et.al.^24^ and includes a correction for the concentration of 1 N)

ΔΔG_DSF_ is the associated finite size discrete solvent correction term^31^;

ΔΔG_NET_ is the correction for periodicity-induced net-charge interactions;

ΔΔG_USV_ is the correction for periodicity-induced undersolvation;

ΔΔG_RIP_ is the correction for residual integrated potential effects.

**Table 3:** Free energy of coupling for ligand-solvent and ligand-receptor system obtained from MBAR method. Total ΔG_bind._

	**Ref**_surface_	**A30T**_surface_	**Ref**_mlddle_	**A30T**_middle_	**Ref**_middle_wat_	**A30T**_middle_wat_	**Ref**_deep_	**A30T**_deep_
ΔG^solv_coul^	-294.6±0.7							
ΔG^solv_vdw^	4.5±0.5							
ΔG^solv(wdv+coul)^	-290.1±0.9							
ΔΔG^DSF_solv^	0.6							
ΔΔG^NET^+ΔΔG^USV^	0.78							
ΔΔG^RIP_solv^	0.13							
ΔG^solv_rest^	-31.1	-30.2	-33.4	-30.8	-32.4	-31.7	-32.0	-31.9
ΔG^prot_restr^	-12.8±0.1	-8.1±0.0	-8.0±0.0	-19.5±0.1	-9.5 ±0.0	-9.4 ± 0.0	-33.2±0.1	-11.4±0.1
ΔG^prot_coul^	-301.3±0.5	-314.0±0.3	-175.8±0.6	-195.8±0.7	-259.5 ± 0.5	-280.8 ± 0.7	-232.8±0.7	-246.5±0.8
ΔG^prot_vdw^	-15.6±0.1	-6.6±0.2	-55.6±0.1	-78.9±0.1	-52.9 ± 0.2	-34.4 ± 0.1	-80.1±0.1	-76.9±0.5
ΔG^prot(wdv+couk+restr)^	-329.7±1.3	-328.8±1.1	-239.5±1.4	-294.2±1.5	-322.0±1.4	-324.5 ± 1.9	-346.0±1.6	-334.9±1.6
ΔΔG^DSF^	18.4	18.5	18.6	18.6	18.5	18.3	18.5	18.4
ΔΔG^NET^+ΔΔG^USV^	0.26							
ΔΔG^RIP^	-4.2	-4.0	-3.7	-3.8	-3.4	-3.4	-4.2	-4.1
ΔG_bind_	4.5±1.6	4.8±1.4	97.7±1.7	40.4±1.7	14.4±1.7	11.0±2.1	-10.9±1.8	0.1±1.8

An infinite-system discrete solvent correction term (DSI) is not included in Table 3 because its value (-73.8 kJ/mol) is the same for protein-ligand and solvent ligand systems and is reset to zero when calculating ΔG_bind_.

In the surface conformation of the ligand, binding is not energetically favorable since in both cases (reference and mutant) the value of the free binding energy is slightly higher than zero. In this conformation, the affinity to HCl*H-His-Rim is the same for both the mutant channel and the reference structure, which is explained by the remoteness of the position of the point mutation A30T from the ligand binding site.

In the case of middle binding without taking into account the solvation of the amino acid part of the ligand, ΔG_bind_ was found to be significantly positive, which suggests that this case is not energetically profitable. At the same time, when the amino acid part of HCl*H-His-Rim is solvated, the free energy of ligand binding to both the reference and mutant proteins decreases, which qualitatively corresponds to the LIE calculation results. In this case, ΔG_bind_ also have close values for the two channel structures, but this conformation, even taking into account ligand solvation, appears to be less energetically favorable than the surface one. According to the free energy calculations, the deep conformation is the most favorable in energy. In this case, according to the data of free dynamics, the water flow in the channel is completely blocked. In addition, in case of deep conformation, the binding of the ligand to the reference is much more advantageous than with A30T, which is consistent with the result of the experiment *in vitro*. In our opinion, this difference may be due to an increase in the volume of the side chain 30T and steric limitations when binding HCl*H-His-Rim to the mutant channel A30T. In addition, the potential barrier which is required to overcome the ligand upon binding in deep conformation appears to increase in the mutant form.

## Conclusion

According to calculations of molecular dynamics, the conformation and position of the ligand in which the imidazole ring of the histidine part of HCl*H-His-Rim is at the level of 30-31 amino acid residues and the adamantane part of the ligand is 0.35-0.4 nm above the plane of imidazole rings H37 is the most energetically favorable and the most stable. In this ligand conformation, the water flow in the channel is completely blocked and binding the ligand to the reference is much more advantageous than with A30T, which is consistent with the result of the *in vitro* experiment. In our opinion, this difference may be associated with an increase in the volume of the side radical 30T and steric limitations upon binding HCl*H-His-Rim to the mutant channel A30T. In addition, the potential barrier which is necessary to overcome the ligand upon binding in deep conformation is apparently increasing in the mutant form.

## Ethical Issues

Not applicable. This article does not describe any studies with human or animal subjects.

## Conflict of Interest

The authors declare that they have no conflict of interest.

## Acknowledgements

The publication has been prepared with the support of the “RUDN university program 5-100”.

This work was supported by the Ministry of Science and Higher Education within the State Assignment Federal Scientific Research Center Crystallography and Photonics of the Russian Academy of Sciences in part of molecular docking and modeling. The calculations were performed by Hybrid high-performance computing cluster of Federal Research Center Computer Science and Control of Russian Academy of Sciences. Available at: http://hhpcc.frccsc.ru).

## Supplementary Materials


Supplementary file 1 contains Tables S1-S2 and Figures S1-S2.
