# Supplementary material for: Studying the Effect of Amino Acid Substitutions in the M2 Ion Channel of the Influenza Virus on the Antiviral Activity of the Aminoadamantane Derivative In Vitro and In Silico
Source: Adv Pharm Bull. 2020 Jul 15;11(4):700–11. doi: 10.34172/apb.2021.079 (PMC8642805; doi:10.34172/apb.2021.079)
Supplement: Supplementary file 1 — contains Tables S1-S2 and Figures S1-S2. [file apb-11-700-s001.pdf]

Supplementary file

Supplementary Table 1. The basic parameters of equilibration steps.

Stage of equilibration	Ensemble	Integration step, fs	Simulation time, ps	Force of non-H atoms position restraints, kJ/mol/nm^2			
				Main chain	Side chain	Lligand	Lipid head
1	NVT	1	25	4000	2000	4000	1000
2	NVT	1	25	2000	1000	2000	1000
3	NPT	1	25	1000	500	1000	400
4	NPT	2	100	500	200	500	200
5	NPT	2	100	200	50	200	40
6	NPT	2	100	50	0	50	0

Supplementary Figure 1. Mass spectrometry on the Bruker UltraFlex II MALDI-TOF time-of-flight mass spectrometer with flexControl 1.1. and flexAnalys 2.2 software for mass spectra acquisition and processing.

H-His-Rim, matrix IAA (3β- indolacrylic acid)

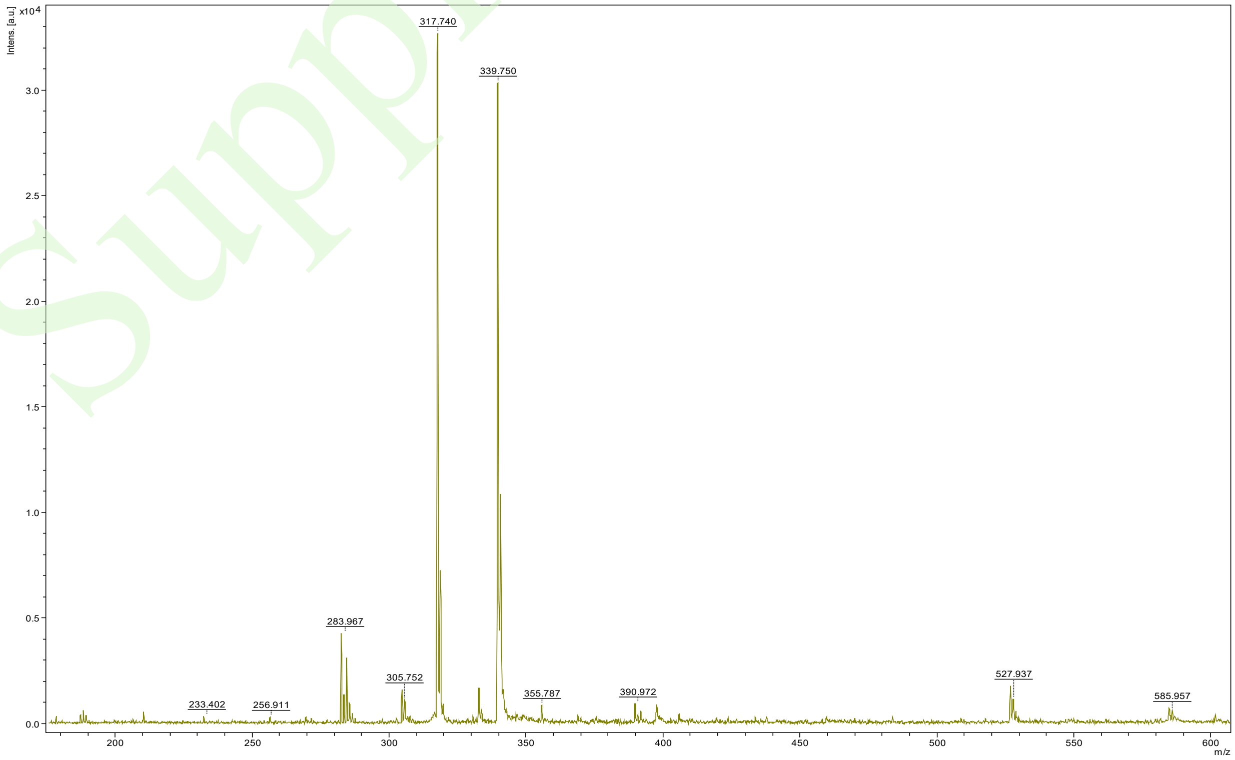

Supplementary

Supplementary Figure 2. Spectral data for the compound HCl*H-His-Rim

NMR C^{13}

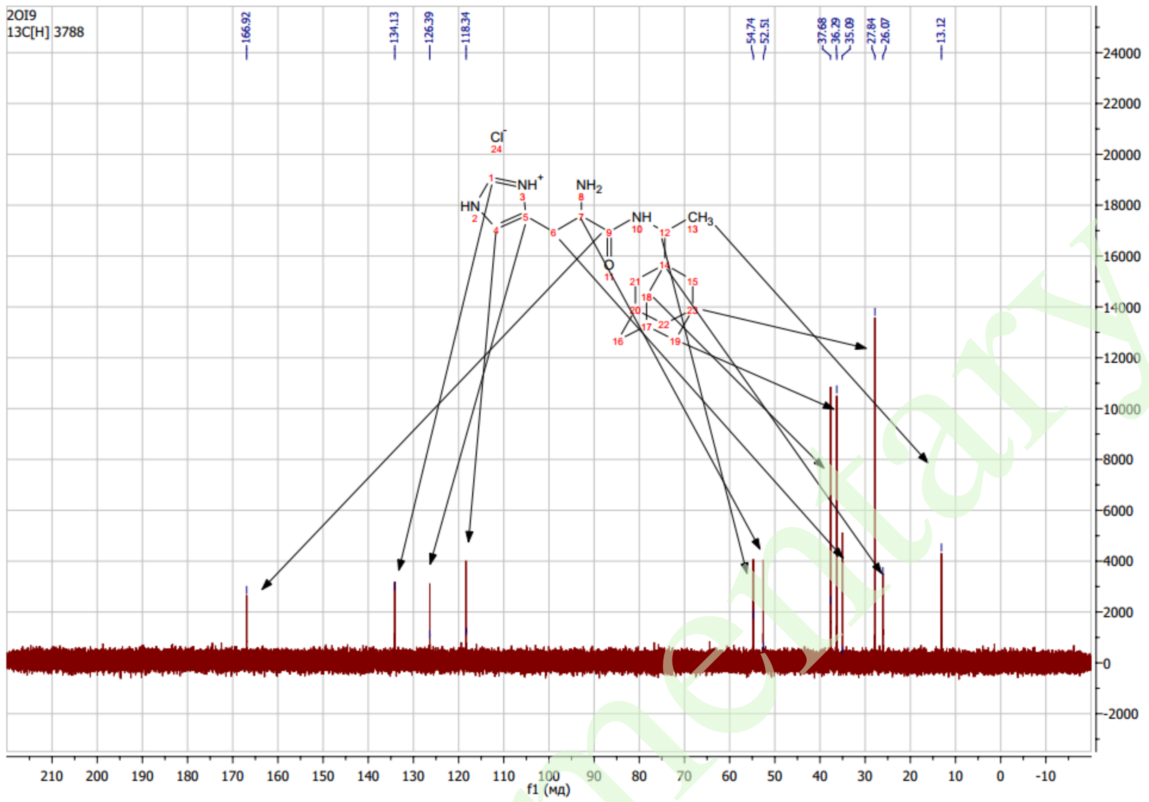

NMR H^1

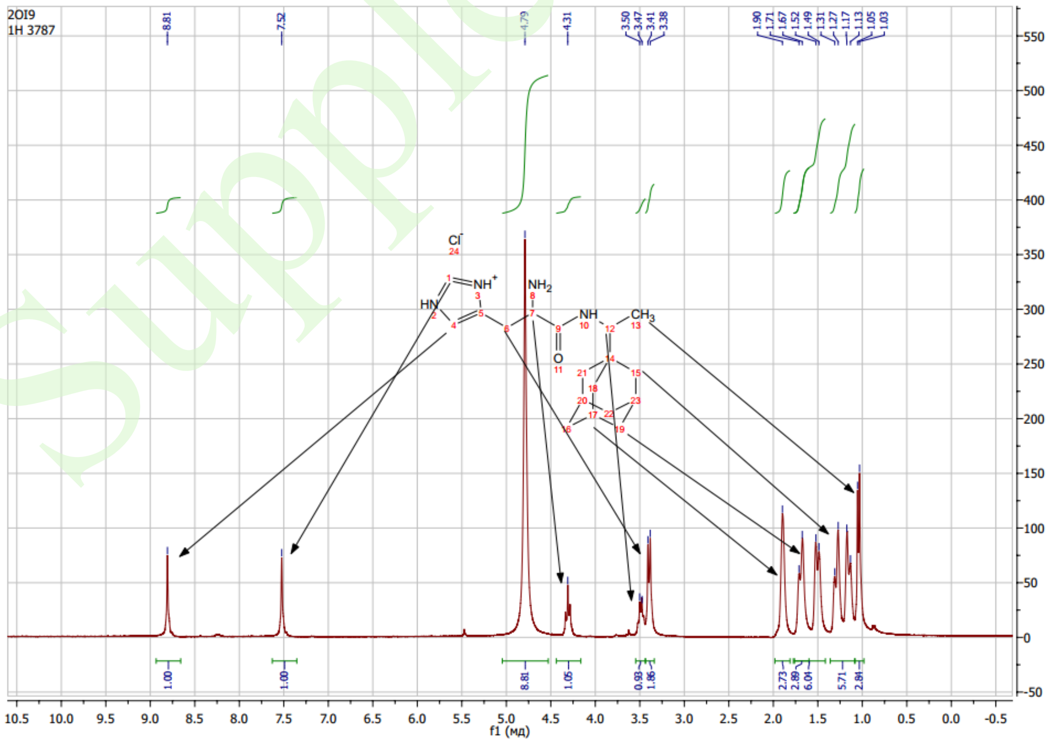

Supplementary

Supplementary Table 2. Linear interaction energy by trajectory slicing.

Protein	Ligand conf.	t_{start}, ns.	t_{end}, ns.	<LIE>, kJ/mol	σ(LIE), kJ/mol	LIE_{min}, kJ/mol	LIE_{max}, kJ/mol
S31N	Deep	0	20	13.1	18.4	-69.0	95.4
	Middle	0	20	54.5	21.6	-13.3	168.3
	Middle	0	1.75	102.7	16.6	49.5	168.3
	Middle	1.8	20	49.8	15.5	-13.3	112.6
	Surface	0	20	-15.2	21.9	-106.3	65.0
S31N_A30T	Surface	0	20	-30.0	22.2	-112.1	58.9
	Deep	0	20	45.7	12.31	-9.8	99.6
	Middle	0	20	39.2	34.1	-71.6	148.1
	Middle	0	10.275	65.8	23.5	5.4	148.1
	Middle	10.325	20	11.0	16.6	-71.6	75.7

Supplementary
